# Development of the adult and child complementary medicine questionnaires fielded on the National Health Interview Survey

**DOI:** 10.1186/1472-6882-13-328

**Published:** 2013-11-23

**Authors:** Barbara J Stussman, Christina D Bethell, Caroline Gray, Richard L Nahin

**Affiliations:** 1 National Center for Complementary and Alternative Medicine, National Institutes of Health, Bethesda, MD, USA; 2 School of Medicine, Child and Adolescent Health Measurement Initiative, Oregon Health & Sciences University, Portland, OR, USA; 3 Palo Alto Medical Center, Health Policy Research Institute, Palo Alto, CA, USA

**Keywords:** Complementary therapies, Complementary medicine, Questionnaire development, Cognitive interviewing, National Health Interview Survey (NHIS)

## Abstract

The 2002, 2007, and 2012 complementary medicine questionnaires fielded on the National Health Interview Survey provide the most comprehensive data on complementary medicine available for the United States. They filled the void for large-scale, nationally representative, publicly available datasets on the out-of-pocket costs, prevalence, and reasons for use of complementary medicine in the U.S. Despite their wide use, this is the first article describing the multi-faceted and largely qualitative processes undertaken to develop the surveys. We hope this in-depth description enables policy makers and researchers to better judge the content validity and utility of the questionnaires and their resultant publications.

## Correspondence/findings

The National Health Interview Survey (NHIS) is an annual in-person household survey of the health of the U.S. civilian, non-institutionalized population conducted by the National Center for Health Statistics (NCHS), Centers for Disease Control and Prevention (CDC). In 2002, 2007 and 2012 supplemental questions on complementary medicine were added to the NHIS. These questionnaires filled a void for large-scale, nationally representative datasets on the out-of-pocket costs, prevalence, and reasons for use of complementary therapies in the U.S. All three surveys were sponsored primarily by the National Center for Complementary and Alternative Medicine (NCCAM), part of the National Institutes of Health (NIH). The first NHIS complementary medicine survey, fielded in 2002, was initiated in-part by the NCCAM 5-Year Strategic Plan 2001–2005, which included the creation of a Special Populations Program to, among other goals, support research on complementary medicine use in racial and ethnic minority populations [1]. In the early 2000’s, existing datasets for complementary medicine use in the U.S. lacked sufficient sample sizes to analyze use by minority populations. Given this deficit, NCCAM made the decision to fund a large national survey of complementary therapies. The NHIS was chosen as the vehicle for the complementary medicine surveys because of its large sample size and its oversampling of non-Hispanic black and Hispanic persons (and Asian persons beginning in 2006).

Analyses of the 2002 and 2007 surveys have resulted in more than 100 publications in peer-reviewed journals [2]. It is expected that the 2012 supplement will have the same significant impact on research and policy-making communities as did the 2002 and 2007 versions, which provided the most comprehensive nationally representative data on complementary medicine use available for the United States. Although many research priorities and specific content identified during the development of the 2002 questionnaire remained as part of the 2007 and 2012 surveys, each questionnaire contained unique topics. For example, the 2012 survey included a series of items related to using complementary therapies for wellness-related reasons. NCCAM decided to include this topic during development of its third strategic plan [3], which placed an emphasis on use of complementary therapies to promote health, wellness and well-being.

This article describes the multi-faceted and largely qualitative approach taken to develop the three questionnaires, particularly detailing the results of expert input and testing of cognitive salience and clarity of questionnaire items. This article will guide researchers and policy makers who plan to use data generated from the surveys, as well as inform quantitative assessments of questionnaire validity and of the research value provided by the resulting datasets.

## Overall development process

The development of the three NHIS complementary medicine questionnaires was not performed within the framework of a planned research study. Instead, questionnaire design and development generally followed steps documented in the literature [4-8] and in an NCHS report on questionnaire design [9]. The NCHS protocol included four phases: 1) planning, 2) exploratory studies, 3) developmental methods, and 4) testing methods. Planning is particularly important for government surveys which generally have tight timeframes, require multiple clearances, and may involve staff from multiple organizations. For each questionnaire, development began 18–24 months prior to data collection in collaboration with survey methodologists from NCHS. The second phase, exploratory studies, is useful for identifying the response process and response problems unique to a given set of questions. This step applies to specific aspects of complementary medicine modalities such as the relationship between self-identity and use of nonvitamin, nonmineral dietary supplements (NVNMDS) [10] discussed later in this article. The third phase, developmental methods, involves iterative rounds of questionnaire evaluation through participant interviews as the main approach for discovering problems with questionnaires. Although the number of respondents need not be large (20–50 respondents), ideally they should differ widely in demographic characteristics to maximize generalizability. The final phase described in the NCHS report, testing methods, refers to large-scale field testing. Although budget constraints did not allow for large-scale field pre-testing, input from field staff was solicited prior to fielding and resultant modifications were made.

Development of the three NHIS questionnaires applied the definition of complementary medicine established by NCCAM: “a group of diverse medical and health care systems, practices, and products not generally considered part of conventional medicine” [1]. Although the specific therapies included varied slightly across survey years, the following were included in all three questionnaires: acupuncture, Ayurveda, biofeedback, chelation therapy, chiropractic care, energy healing therapy, hypnosis, massage, naturopathy, NVNMDS, homeopathic treatment, diet-based therapies, yoga, tai chi, qi gong, and meditation and other relaxation techniques. The 2002 questionnaire collected complementary medicine use for adults only, whereas the 2007 and 2012 included children and adults. All three surveys were approved by the NCHS Research Ethics Review Board.

Specific steps detailed in this article include: (a) literature reviews, (b) feedback from scientific and practitioner communities, (c) expert input and workshops, and (d) participant interviews (cognitive testing). Additionally, for the 2012 child questionnaire, quantitative analysis of data from the 2007 child complementary medicine questionnaire (frequencies analysis of prevalence of therapies by age, race/ethnicity, disease status, and use for health conditions) and focus groups with health professionals and the lay public were conducted. Although these steps took place sequentially within the overall development process (literature review, then expert panels, then draft initial questions, then cognitive testing, then refinement), the synthesis of these components to yield the final content was an iterative process. For example, after the expert panels, during the drafting of initial questionnaire items, we often discussed a particular series of questions with the expert panel member who focused the corresponding discussion during the meeting. Likewise, while refining the questionnaire based on results of cognitive testing, we often referred back to notes from workshops, consulted with expert panel members, or sought additional research articles to decide on the most appropriate solution. Specific steps used in the development of each questionnaire varied (Table 1) and are described in discrete terms below.

**Table 1:** Iterative development process for complementary medicine questionnaires

**Questionnaire development steps**	**Year**		
	**2002**	**2007**	**2012**
Literature review	X	X	X
Feedback from scientific communities on previous questionnaires		X	X
Expert input/panel	X		X
Conceptual workshop			X
Inductive cognitive review			X
Cognitive interviews with staff members			X
Cognitive interviews with volunteers from general public	X	X	X
Focus groups with health leaders and subject matter experts			X
Feedback from Census Bureau interviewers	X	X	X
Quantitative analysis of previous child survey data			X

## Literature reviews

The goal of each literature review was to identify previous surveys on complementary medicine use in order to help determine which therapies to include in the NHIS questionnaire. These surveys were reviewed by the expert panels when making recommendations to NCCAM and NCHS staff on what complementary therapies should be included in the NHIS. Literature reviews for all three surveys employed the search paradigm utilized by Wootton and Sparber [11]. Development of the 2002 questionnaire began by reviewing smaller-scale national and regional surveys identified by Wooton and Sparber through January 5, 2001. Development for the subsequent surveys examined U.S. national or regional surveys on complementary medicine that were published *after* January 5, 2001. Only U.S. surveys were considered during the literature review since the terminology, definitions and regulations vary considerably from country to country. For instance, although in the U.S. dietary supplements are regulated as food-based in the Dietary Supplement Health and Education Act [12], in other countries, such as Germany, they are often regulated as drugs [13] or, as in Japan, covered by national health insurance [14].

### 2002

The systematic review by Wooton and Sparber [11] identified seven population-based surveys published in peer-reviewed journals through January 5, 2001. Of these, five reported data from national population-based surveys on complementary medicine use in the U.S. [15-19] and two reported data from state-level population-based surveys – Florida [20] and South Carolina [21]. The report by Druss and Rosenheck [19] was based on data from the 1996 Medical Expenditures Panel Survey supported by the U.S. Agency for Healthcare Research and Quality. These seven surveys served as a starting point by the expert panel for identifying content to be added to the 2002 NHIS. Item wording from these surveys was often changed to be consistent with the NHIS format.

### 2007

In preparation for the 2007 NHIS complementary medicine survey an updated search of the scientific literature published up to January 2006 was conducted, and five additional national population-based surveys in adults [22-26] and one state-level - Michigan [27] were identified. Two of these were initial reports from the 1999 and 2002 NHIS surveys [22,24]. When reviewing the four non-NHIS surveys, particular attention was paid to complementary approaches not included in the NHIS surveys, as well as to reasons individuals said they used complementary therapies. Among the complementary therapies included on these surveys but not on the 2002 NHIS were traditional healers and movement therapies, both of which were added to the 2007 survey.

### 2012

From published reports through January 2011, the search update for the 2012 NHIS identified three new national-population based surveys [28-30], three state-level population-based surveys – California [31], Hawaii [32], and Minnesota [33] - and one survey of active duty military personnel in the U.S. Navy and marine corps [34]. These were reviewed for complementary approaches not contained in the 2007 NHIS, as well as wellness-related reasons individuals used these therapies. No previously unidentified complementary therapies were found in these surveys.

In addition, for the 2012 questionnaire, literature searches were employed specifically to inform questionnaire design related to reasons and motivations for using complementary therapies, such as for wellness or well-being, as well as questions asking about insurance coverage [35-40]. PubMed and Google Scholar were searched, and additional references were suggested by outside experts attending a workshop on wellness (Section Wellness workshop) and members of a think tank (Section Research topic think tank) to identify research priorities for the 2012 questionnaire. Wellness-related reasons identified in the literature for why individuals use complementary therapies include physical, spiritual, and emotional well-being, nutrition and lifestyle, and to avoid pharmaceuticals [35-40].

To identify additional dietary supplements for inclusion in the 2012 NHIS, dietary supplement sales data were reviewed [41]. Among the many products added to the NHIS survey were Acai, bee pollen and other bee products, digestive enzymes and methylsulfonylmethane (MSM). In total, 119 nonvitamin, nonmineral dietary supplements were queried in the 2012 NHIS versus 44 in the 2007 NHIS.

#### Feedback from scientific and practitioner communities

Both the 2002 and 2007 surveys received considerable attention by the public, clinical and research communities. Staff at NCCAM received unsolicited feedback from complementary medicine researchers and practitioner organizations that was used to inform the development of the 2007 and 2012 surveys. Much of this feedback came during meetings held for other purposes and was not collected systematically. Because this feedback was unsolicited and non-systematic, data was not kept on who provided specific feedback and when.

### 2007

Based on unsolicited feedback on the 2002 questionnaire and updated literature reviews, the following changes were made for the 2007 survey: 1) Addition of questions on complementary medicine use by children and the condition being treated by these therapies; 2) addition of out-of-pocket expenditures on complementary therapies; 3) addition of movement therapies (Feldenkrais, Alexander technique, Pilates, Trager psychophysical integration); 4) addition of traditional healers to better capture complementary medicine use in Hispanic and Native American populations; 5) deletion of the megavitamin section; 6) addition of questions to find out why some adults did not use five types of complementary therapies - acupuncture, chiropractic or osteopathic manipulation, NVNMDS, yoga, and meditation - chosen based on the most commonly used therapies from each of the major categories of complementary medicine as defined by NCCAM at the time of questionnaire development [1]; 7) deletion of folk medicine; and 8) addition of questions about the relationship between complementary and conventional treatments (adult version only). Content for 2002, 2007, and 2012 questionnaires are shown in Table 2. Other changes, such as the addition of questions on vitamins and minerals, and an expanded section on NVNMDS, grew out of consultations with other parts of the NIH.

**Table 2:** Content of 2002, 2007, and 2012 NHIS complementary medicine questionnaires

**Topic**	**2002**	**Enhancements/changes for 2007**	**Enhancements/changes for 2012**
Practitioner-based therapies included in survey	Acupuncture	Chiropractic care replaced with chiropractic or osteopathic manipulation	Added craniosacral therapy
		Rreiki no longer distinguished from other energy healing therapies	Modified list of traditional healers
	Ayurveda	Added movement therapies (Feldenkreis,	Allowed participants to report whether chiropractor or osteopathic physician provided manipulation therapy
		Alexander Technique, pilates, Trager	
	Biofeedback	Psychophysical integration)	
		Added Traditional Healers (Curandero, Espirtista,	
	Chelation therapy	Hierbero or Yerbera, Shaman, Botanica,	
		Native American Healer, Sobador)	
	Chiropractic care	Removed folk medicine	
	Energy healing therapy/Reiki		
	Folk medicine (such as Curanderismo or Native American healing)		
	Hypnosis		
	Massage		
	Naturopathy		
Self-care therapies included in survey	Nonvitamin, nonmineral dietary supplements (NVNMDS) (35 total)	Included 44 NVNMDS on flashcard	Pared down set of questions on vitamin and mineral supplements
	High dose or megavitamin therapy	Added vitamin and mineral supplements	Expanded list of NVNMDS to 21 on flashcard and 98 in lookup table (119 total)
	Homeopathic treatment	Removed high dose or megavitamin therapies	Classified meditation into 3 broad categories (mantra, mindfulness, and spiritual)
	Special diets (vegetarian, macrobiotic, Atkins, Pritikin, Ornish, zone)	Added South Beach to special diets	Removed breathing exercises as separate therapy but included it within other therapies (hypnosis, biofeedback, meditation, guided imagery, progressive relaxation, yoga, tai chi, qi gong)
	Yoga, tai chi, qi gong	Pared down questions on prayer for your own health	
	Relaxation techniques (meditation, guided imagery, progressive relaxation, deep breathing exercises)		Removed zone and South Beach from special diets
	Prayer for your own health		Removed prayer for your own health
Information collected for each therapy	12 month prevalence	Added lifetime prevalence	Added whether all or some of the costs were covered by health insurance
	Frequency of use in past 12 months for practitioner-based therapies	Added frequency of use in past 12 months for self- care therapies	
			Modified questions on out-of-pocket costs to allow for different ways of reporting
	Use for specific health problem or condition	Added out-of-pocket costs in past 12 months (per visit/purchase)	Cost of self-help materials expanded to include both practitioner-based and self-help therapies
	Reasons for use (conventional care would not help, conventional care was too expensive, combination of conventional care and complementary medicine would help, recommended by conventional provider, thought it would be interesting to try)	Added whether also using conventional care for the same health problem as using complementary therapy	Narrowed questions on reasons for use to the 3 therapies most important for health; did not ask for chelation and Ayurveda; asked collectively for movement, traditional healers, and special diets
		Added additional reasons for using complementary therapy (energy, wellness, immune function, recommended by family, friends, or coworkers)	Greatly expanded reasons for use to include wide range of wellness-related reasons, motivators, and outcomes
	How much therapy helped health problem		Added how much therapy helped most important reason for using it
	Importance of use of therapy		Added how much use of therapy helped the specific health problem
	Disclosure to conventional provider		
	Whether insurance covered any of the Costs		
			Added importance of use of therapy
			Modified disclosure to apply to personal care provider
			Added reason for not disclosing use
			Added sources of information about therapy
Additional information collected	Numerous health conditions added to NHIS core	Added additional health conditions to NHIS core	Modified list of health conditions added to NHIS core based on prevalence data from prior surveys
		Collected detailed follow-up information for up to two NVNMDS	Added questions about having a personal health care provider and whether he/she is the same practitioner for complementary therapies
		Expanded list of reasons for using NVNMDS and vitamin and mineral supplements	Expanded child questionnaire to be almost identical to adult; limited to children age 4+
		Added reasons didn’t use certain therapies (acupuncture, chiropractic, NVNMDS, yoga, and meditation)	
		Added child questionnaire with subset of items from adult survey (12 month prevalence, use for specific health problem, use of dietary supplements for sports)	

### 2012

Based on unsolicited community feedback about the 2007 survey several items were modified for the 2012 version. For example, follow-up questions on yoga were restricted to only those using meditation and/or breathing exercises as a component of yoga. Additionally, the list of NVNMDS was expanded beyond that of 2007.

## Expert panels and workshops

Expert panels were convened for the development of the 2002 and 2012 surveys, but not for the 2007 survey. Panel members were invited based on their published work and expertise, and were considered experts in their own field. No sampling method was utilized. For each of the three in-person panels described below an open-dialogue process was used, with iterative summaries of high level input and priorities set throughout the meetings. The 2002 panels consisted of half-day phone calls and numerous email exchanges, while the 2012 panels were day-long meetings. Each meeting began with introductions, background presentations and an explanation of goals and objectives for the session. Input was solicited from each member of the panel and careful attention was given to keep discussions focused and on-target. Time was allocated at the end of each meeting to reach consensus on conclusions discussed throughout the meeting. For the in-person 2012 panels, scientific notetakers were utilized to ensure thorough documentation.

### 2002

In February 2001 a panel was convened made up of 13 national experts in survey design and complementary medicine research and practice, along with NCCAM and NCHS staff, to prioritize content for the 2002 questionnaire. A second panel made up of 13 experts in women’s health, mental health in minority populations, complementary medicine use by African Americans and Native Americans, use of Chinese medicine, and cultural relevance was assembled to review draft questionnaire items. Table 3 shows a complete listing of expertise on each panel. The second panel did not convene as a group, but expert opinion was solicited in writing from each individual member related to the person’s area of expertise and additional modifications were made based on this input.

**Table 3:** Expertise of panel members

**2002**		**2012**	
**Initial development**	**Minority population**	**Wellness workshop**	**Research topic think tank**
**(n = 13)**	**(n = 13)**	**(n = 27)**	**(n = 24)**
Epidemiology (n = 2)	Aging, women’s health and use of complementary therapies	Aging, dementia, and health rehabilitation (n = 4)	Pediatrics and complementary therapies (n = 2)
Health policy	Diabetes and complementary therapies	Chronic disease and health behaviors (n = 3)	Healthcare utilization and complementary therapies (n = 2)
Health psychology	Ethnic and racial minorities and complementary therapies	Developmental disabilities, and parent and child behaviors	Pain conditions. epidemiology and complementary therapies
Health services research	Health outcome research in Hispanic populations	Health education and complementary therapies	Health services research and complementary therapies (n = 2)
Minority populations and complementary therapies	Healthcare disparities	Health outcomes research	Public health, Hispanic populations, and complementary therapies
Nutrition and physical activity	Minority populations and complementary therapies	Health psychology and spirituality	Quantitative psychology and complementary therapies
Pain conditions and complementary therapies	Public health and African Americans	Human values and moral development	Health Psychology and complementary therapies
Public health (n = 2)	Public health and the environment	Patient reported outcome measures	Psychometrics
Questionnaire design	Use of complementary therapies among African Americans	Nephrology, clinical research, and complementary therapies	Health education and complementary therapies
Sociology	Use and effectiveness of complementary therapies in health and illness	Pain conditions, epidemiology and complementary therapies	Geriatrics, rural healthcare, and complementary therapies
Survey design and complementary therapies	Women’s health and complementary therapies (n = 3)	Pediatric psychology and illness (n = 2)	Health economics and complementary therapies
		Personality	Analysis of complex datasets
		Positive psychology	Complementary therapy use among racial and ethnic minorities
		Quality of life research	Survey Methodology (n = 2)
		Questionnaire design, survey methodology and health statistics (n = 3)	Medical Care statistics
		Social and psychiatric epidemiology and social support	Naturopathy (n = 2)
		Social Psychology and mental health	Sociology and qualitative research
		Substance abuse	Survey design (n = 2)
		Wellness, and psychological issues of dealing with chronic illness	

The first expert panel suggested specific therapies to include in the 2002 NHIS. The expert panel also identified four areas of research priority: 1) Prevalence of complementary medicine use; 2) purpose of complementary medicine use (whether for a specific condition, general wellness, or both); 3) disclosure of complementary medicine use to conventional providers; and 4) inventory of natural supplements and prescription medications (not fielded due to logistical reasons). The highest priority was to identify the specific conditions or diseases individuals report using complementary therapies to treat, which is distinct from assessing the prevalence of complementary medicine use among individuals who experience one or more health conditions. Analysis of this later construct is possible by linking the NHIS complementary medicine questionnaires to other NHIS core questionnaire data files. Additional conditions and diseases hypothesized to correlate with complementary medicine use were added to those already in the NHIS core questionnaire. The second panel identified ways to improve the cultural sensitivity of the questionnaire. For example, the use of the word “traditional” to refer to Western medical practices was replaced with “conventional” based on the recommendation from a cultural expert concerned that “traditional” has different meanings to individuals with regard to various culturally related practices.

Based on review of the literature and input from the panels of outside experts, a questionnaire was constructed, edited and refined by NCHS and NCCAM staff. Underlying this process was a set of criteria for questionnaire development identified from the literature [42-44] (Table 4). Once the questionnaire was constructed, the expert panels were given an opportunity to provide additional feedback. The draft questionnaire was also circulated to experts throughout the NIH for comment.

**Table 4:** **Criteria used to develop items for complementary medicine questionnaires**^**1**^

1 Literacy level of questions at 8^th^ grade or below	
2. Specific questions are generally easier to answer than broad ones	8. Questions covering multiple concepts are decomposed into single questions
3. Question captures what researcher intended (i.e. every respondent answering the same question)	9. Questions are designed to avoid social desirability effects
4. Question interpreted as intended by persons in a range of socio-demographic groups	10. Avoid:
	a. questions worded in the negative
5. Response categories fit the question and are non-overlapping, clear and unambiguous	b. complex questions
	c. questions combining two items in one (double barreled)
6. Reference periods are clear and match respondent’s ability to recall	d. jargon
	e. ambiguous terms
7. Terms and definitions are defined	f. questions that lead respondents toward a particular answer

^1^This criteria were identified from the literature: References [42-44].

### 2012

#### Wellness workshop

As evidenced in NCCAM’s strategic plans, the Center’s priorities have evolved during the past decade from a focus on treatment of disease to symptom management and promotion of optimal health [1,3]. In August 2009, NCCAM staff sponsored a one-day workshop on how to best capture the concept of wellness and optimal health via survey items. Twenty-seven individuals participated in the workshop including behavioral scientists from universities and non-profit organizations, as well as representatives from NCCAM and other parts of NIH, CDC, and the Food and Drug Administration (Table 3). Because it was the sole internationally accepted definition at the time, NCCAM utilized the World Health Organization’s definition of wellness to generate discussion: “wellness is the optimal state of health of individuals and groups. There are two focal concerns: the realization of the fullest potential of an individual physically, psychologically, socially, spiritually, and economically, and the fulfillment of one’s role expectations in the family, community, place of worship, workplace and other settings” [45]. In preparation for the workshop, the planning committee identified criteria to help guide discussion [46-56].

Workshop attendees discussed several topics including how best to define wellness and differences between wellness and well-being. Discussion at the workshop focused on psychological, physical, spiritual, and social measures of wellness, as well as the relationship between wellness and use of complementary therapies. Workshop results guided the development of wellness items for the 2012 survey, particularly the interviewer protocol for cognitive testing of the wellness concept.

Interviewer protocols are guides to assist in the conduct of qualitative interviews and generally contain verbal probing and follow-up questions to aid in providing some standardization across interviews [57]. The interviewer protocol used for the testing of the wellness concept contained questions to elicit reasons or motivations for using complementary therapies without presupposing a “treatment” reason and respondents were asked about changes noted in “mind or body” when using the therapy (see section Concepts of wellness and well-being).

#### Research topic think tank

In September 2010 NCCAM staff convened a panel of experts to review the previous two NHIS complementary medicine questionnaires and identify items to retain as well as key research areas not previously covered. The panel was comprised of 24 individuals with expertise in health services research, minority health, economics, and psychometrics, as well as complementary therapies (Table 3). Based on feedback from the research topic think tank, an extensive set of questions was included in the draft questionnaire about insurance coverage and out-of-pocket expenses. This draft set of questions captured detailed information about deductible amounts, number of visits and other dollar amounts covered by insurance, and whether the respondent’s complementary medicine use was reduced due to limited insurance coverage. This section was ultimately pared down due to complexity and time restraints (see Section Draft questionnaire).

Members of the think tank also recommended that deep breathing be removed as an independent intervention in the survey given the wide variability in how participants interpret the question as identified during cognitive interviews. Instead, queries on the use of deep breathing exercises were imbedded as follow-up questions for participants using yoga, tai chi, qi gong, meditative techniques, hypnosis and biofeedback. Finally, the think tank recommended that the survey provide some specificity on the types of meditation being used. Thus, instead of asking a global question as to whether a participant used meditation, the 2012 survey asked about three broad classes of meditation identified in an Agency for Healthcare Research and Quality systematic review: mantra, mindfulness and spiritual [58].

NCCAM staff members reviewed successive drafts for face validity and item phrasing, and an NCCAM staff member trained in cognitive interviewing techniques administered the draft questionnaire to nine NCCAM staff members with various cultural and academic backgrounds to better refine the questionnaire before it underwent cognitive testing with volunteers from the general public.

## Cognitive interviewing

Cognitive interviewing, a technique to learn respondents’ thought processes as they answer survey questions, has become standard practice for survey development [59]. The aim is to uncover how respondents interpret the intent and meaning of survey questions, and whether or not these match those of the researcher. The interview generally involves one interviewer and one respondent, usually takes between 30 to 90 minutes, and respondents are often compensated for their time. The interviewer will read the draft questions to the respondent and then use probing techniques to determine how the respondent is interpreting the questions and what the respondent is thinking while answering. The interviews were videotaped and their verbal content entered into Q-notes, a software system created by NCHS’s Questionnaire Design Research Laboratory (QDRL) staff to organize and help with the analysis of cognitive interview data. Interpretations of key terms and concepts in each question were examined and compared among participants in order to identify inconsistent interpretations. Ideally, interviewing continues until “saturation” is reached, that is, no new data are emerging and all the concepts are well defined and explained [60]. However, because the NHIS must begin data collection in January each year, real-world deadlines did not allow for saturation for all sections of the questionnaire; rather, cognitive interviewing focused mainly on new topic areas added in each survey year, or existing sections that were substantially expanded (e.g. NVNMDS in 2007).

After receiving IRB approval, QDRL staff used a variety of recruitment methods to obtain volunteers for cognitive testing including advertisements in the Washington Post and alternative medicine magazines, flyers placed at various co-ops and yoga studios, and the offices of various providers of complementary therapies. Informed consent was obtained from all participants prior to the start of the interview. For each survey year, a total of 25–48 in-depth semi-structured or open-ended interviews were conducted with volunteers from the general public with a range of socio-demographic characteristics (Table 5). During these interviews, questionnaire concepts were tested for cognitive salience and clarity. Possible actions taken as a result of cognitive testing included accepting the original question, accepting the original question with minor edits, accepting the original question with major edits, dropping the original question, and writing a new question (Table 6). The questionnaire development process was fluid and iterative so that changes were made and the questionnaire re-tested throughout the development process. Table 7 provides additional examples of problems found during cognitive review of all three survey questionnaires and the resulting modification made in order to reduce response error.

**Table 5:** Demographic characteristics of participants in cognitive interviewing, 2002, 2007, 2012

	**2002 (n = 25)**	**2007 (n = 32)**	**2012 (n = 48)**	
	**Draft Q**	**Draft Q**	**W-being (n = 24)**	**Draft Q (n = 24)**
**Age**				
Under 35	†	11	6	3
35+	†	21	18	21
**Gender**				
Female	20	18	11	16
Male	5	14	13	8
**Race/Ethnicity**^**1**^				
Hispanic	0	2	8	0
Non-Hispanic White	17	8	12	9
Non-Hispanic Black	7	20	4	13
Asian	1	1	2	2
Other	0	1	6	0
**Education**				
High school or below	8	9	0	0
More than high school	17	23	24	24
**Income**^**2**^				
Under $20,000	5	13	4	6
$20,000+	19	18	20	18
**Employed**				
Yes	†	†	19	14
No	†	†	5	10
**Marital status**				
Never married	†	†	11	10
Currently married	†	†	7	5
Separated	†	†	2	1
Divorced	†	†	4	6
Widowed	†	†	0	2

^†^Data unavailable.

^1^The total for the 2012 race/ethnicity category is greater than the number of participants for 2012 because the data were collected such to allow overlap between Hispanic and other categories.

^2^1 participant in 2002 and 1 participant in 2007 had unknown income data.

**Table 6:** Examples of actions taken based on results of cognitive review and focus groups, 2012

**Question**	**Action taken**	**Justification**
*Did you see a practitioner for/use [therapy] because it was recommended by a medical doctor?*	Accept original question	Cognitive testing confirmed that respondents were primarily thinking of “Western,” “mainstream” medical doctors when they responded to this question, as intended.
Original question: *Did you do breathing exercises as part of [certain therapies]?*	Accept original question with minor edits	Testing revealed that respondents understood “breathing exercises” differently so that some thought it included merely taking a deep breath whereas others thought it was a more formal technique. Therefore, a definition was included in the text of the question.
Final question: *Did you do breathing exercises as part of [certain therapies]? Breathing exercises may involve actively controlling the way air is drawn in, or the rate or depth of breathing.*		
Original question: *During the past 12 months, did you see practitioner for/use [therapy] for any of these reasons…”*	Accept original question with major edits	This question was divided into two questions and re-worded so that only respondents using medical treatments were asked whether experiences with medical treatments influenced their use of complementary therapies, and the type of medical treatment is explicit. Cognitive testing of the original question revealed that respondents found the reference to “medical treatments” unclear. For respondents who were not receiving or using any medical treatments, these questions appeared not to be applicable. In fact, a number of respondents specifically commented that these questions were not relevant to their circumstances.
*…Medical treatments were not helping you?*		
*…Medical treatments were too expensive?*		
*…[modality] combined with medical treatments would help you?*		
Revised and restructured into 2 new questions:		
*Did you receive any of the following medical treatments for*		
*[condition using complementary therapy for]…”*		
*Prescription medications?*		
*Over-the-counter medications?*		
*Surgery?*		
*Physical therapy?*		
*Mental health counseling?*		
[next question asked for respondents who said yes to any items in question above]?		
*DURING THE PAST 12 MONTHS, did you see a practitioner for/use [modality] for any of these reasons…*		
*These medical treatments do not work for the health problem you want to treat or prevent?*		
[next question asked for respondents using prescription medications and/or over the counter medications]		
*Prescription medications/Over the counter medications cause side effects?*		
*Do you currently see a practitioner for [therapy] more, less or about the same as you did one year ago?*	Question dropped	Question was problematic for respondents who see a practitioner only sporadically or on an “as needed” basis. For instance, one respondent who had used homeopathic treatment in the past 12 months said she only uses it when she has a flare up and therefore found it difficult to compare her current use to her use 1 year ago. Similarly, a respondent who did not practice yoga with any regularity had difficultly answering.
*Original question: During the past 12 months, did you see a practitioner for/use [therapy] because it is how you were raised?*	New question written	Although the meaning is similar, this item was re-worded to better capture the way some respondents are influenced to use complementary therapies by cultural factors. When hearing the original question, several respondents hesitated and seemed to find the original wording awkward, evidenced by one respondent who said, “That sounds very odd.” The phrasing “how you were raised” suggested a more deliberate action than merely being exposed to complementary therapies at an early age. “It was part of your upbringing” better captured unintentional or cultural exposure which was the intent of the item.
*Final item: During the past 12 months, did you see a practitioner for/use [therapy] because it was part of your upbringing?*		
General wording of "disease or health problem" found in many questions	Modified	Changing wording to "health problems, symptoms, or conditions" is more comprehensive and inclusive
DURING THE PAST 12 MONTHS, did you or another family member get information about [fill1: modality] from any of the following sources?	Question written (didn’t exist previously)	Information sources about complementary therapies was identified as an important and missing topic during focus groups
The internet?		
Books, magazines, or newspapers?		
Television or radio?		
Scientific articles?		
Health food stores?		
[fill1: Not including the practitioner [fill: S.C. name] saw for [fill2: modality] DURING THE PAST 12 MONTHS, did you let [fill S.C. name]’s personal health care provider know about [fill: his/her] use of [fill3: modality]?	Question written (didn’t exist previously)	Communication about complementary medicine use with other health care providers was identified as an important and missing topic during focus groups
If no, why not:…		
[fill: S.C. name] was not using it at the time?		
They discouraged use of it in the past?		
You were worried they would discourage it?		
You were concerned about a negative reaction?		
You didn’t think they needed to know?		
They didn’t ask?		
You don’t think they know as much about it as you do?		
They didn’t give you enough time to tell them?		

**Table 7:** Examples of response error in cognitively tested questions for 2002, 2007, and 2012 questionnaires

**Questionnaire item**	**Problem identified during cognitive testing**	**Type of error**	**Resolution**
Have you ever used high dose or megavitamin therapy for your own health or treatment?	Many respondents who took vitamin supplements and/or a daily multi-vitamin responded “yes” even though they were not taking megavitamins.	Misinterpretation	Question divided into two parts so respondents who took any kind of vitamins had a chance to say “yes.” Second question screened out the non high-dose users.
(2002)			
*Have you EVER seen a provider or practitioner for movement therapies?*	This question posed some definitional problems. Respondents had different definitions of movement therapy, including yoga and, more commonly, physical therapy.	Definition unknown	Specific movement techniques (Feldenkreis, Alexander Technique, Pilates, Trager Psychophysical Intergration) were asked about individually
*(2007)*			
*How old where you when you first saw a practitioner for [therapy]?*	It was nearly impossible for persons who were heavy users of complementary therapies for a long time to remember the age at which they started using various complementary therapies.	Failure to recall	Question dropped
*(2007)*			
*Have you EVER used natural herbs or other non-vitamin supplements for your own health?*	For some people, using herbal, non-vitamin supplements carries meaning beyond simply using an herb for a specific purpose. As one respondent put it, using alternative therapies is a “way of life.” As a result, people who do not see themselves in this light define their supplement use differently from those they view as “users” of supplements. Several respondents who had no complementary therapy identity wanted to be sure they weren’t labeled as “users.” And this concern affected the way they interpreted and answered the questions. In the end, false negatives were elicited from respondents with this perspective; that is, the question is not capturing all people who use herbal supplements. Furthermore, the data suggest herbal users may not be missed at random, representing a pattern that would lead to biased estimates [62].	Answer based on self-concept rather than actual behavior	Question limited to specific pills, capsules, tablets, or liquids labeled as a dietary supplement, listed on a flashcard shown to the respondent
*(2007)*			
*DURING THE PAST 12 MONTHS, did [child] pray specifically for the purpose of his/her OWN health?*	This question asked respondents about something they have no way of knowing for sure (“I don’t know”; “I don’t think so”; “I’m guessing no”; “not that I’m aware of”). Those with small children can make a better assessment because prayers are sometimes said out loud together, but those with older children usually have to guess [62].	Information unknown	Question dropped
*(2007)*			

### 2002

Twenty-five in-depth semi-structured interviews were conducted; except for chelation therapy, collectively, interviewees had used every complementary medicine identified by literature review and outside experts for inclusion in the questionnaire. Two main findings quickly emerged: 1) modalities were practiced differently with respect to seeing a practitioner or using on one’s own; and 2) many of the initial modalities tested were not discrete and overlapped with other modalities tested. To mitigate the first problem, NCHS staff, with input from the expert panel, identified 10 therapies that are mainly practitioner-based. For these therapies, the scope was narrowed to ask “Have you ever seen a provider for…” rather than “Have you ever used…” This better matched the way respondents described using complementary therapies during interviews. To reduce overlap among therapies, “Chinese medicine” was dropped because it was found to encompass many of the more specific therapies such as acupuncture and NVNMDS. In subsequent versions of the questionnaire, “home remedies” was tested as a self-care therapy and was deleted because it was found to mean different things to different respondents, as well as overlap with NVNMDS, vitamins, and homeopathy. Ultimately, a wide range of modifications and restructuring took place based on the results of cognitive testing including: 1) practitioner-based modalities were separated from self-use modalities; 2) similar modalities were consolidated (e.g. yoga, tai chi, and qi gong were combined) or deleted (e.g. Chinese medicine); 3) questions that lacked meaningful responses or consistent interpretations were deleted (e.g. age of first use, “bad reactions,” and “home remedies.”) [61].

### 2007

Thirty-two cognitive interviews were conducted on seven different versions of the 2007 draft questionnaire. Results from testing reaffirmed two issues that surfaced during cognitive testing of the 2002 questionnaire. The first relates to home remedies, which was re-tested for the 2007 questionnaire as a practitioner-based therapy, with the goal of capturing visits to complementary medicine practitioners in minority and ethnic populations. However, testing revealed that respondents often do not consider home remedies to be practitioner-based, even when the question text includes the phrase “Have you ever seen a practitioner for…” Although respondents spoke of both home remedies and folk medicine as practices passed down through the family, as found during cognitive interviewing for the 2002 questionnaire, the two terms meant different things to different participants and were therefore deleted. Second, heavy users of complementary therapies had difficulty recalling how old they were when they first used a particular therapy even when provided age categories. Because testing showed that estimates would be less reliable for long-time users of complementary therapies compared to recent users, the question was deleted [62].

In addition to these findings, cognitive testing also revealed that the question on “deep breathing exercises” produced numerous false positive responses. Many respondents had very little knowledge of this technique and essentially guessed at its meaning. One respondent said yes to having used deep breathing because “this is when you let out a sigh of being home to get your second wind to cook dinner.” [62]. To try to mitigate this problem, a definition was added to the term, but it was ultimately dropped from the 2012 survey based on Think Tank recommendations (Section Research topic think tank). Particular attention was also given to three sections that were new and/or expanded from the 2002 questionnaire (see below).

#### Child section

For the 2007 NHIS, NCCAM expanded the questionnaire to include a limited set of items on complementary medicine use by children, a topic that had not been previously studied on a national level. The NHIS child section is answered by proxy, usually the child’s parent. As expected, testing revealed that parents do not always know the answers to items on motivations and behaviors related to their children, such as whether a child prays for his or her health. Questions on motivations and prayer were, therefore, not asked in the child section.

#### Mineral and NVNMDS sections

Based on interest and funding by the NIH Office of Dietary Supplements, the 2007 questionnaire included expanded sections on vitamin and mineral supplements and NVNMDS. These sections collected detailed follow-up information for up to two specific vitamins or minerals or NVNMDS supplements and an expanded list of reasons for using these. Although the sections on vitamins and minerals and NVNMDS were identical, cognitive testing revealed problems unique to the NVNMDS items [10,62]. First, it became apparent that respondents lacked a consistent, agreed-on definition of an herbal supplement. Respondents held wide-ranging and varying definitions of NVNMDS and efforts to craft a definition that met researchers’ criteria and was understandable to respondents fell short. Ultimately, the best approach was to simply ask respondents about taking specific supplements listed on a flashcard. This made the survey response task concrete and less reliant on shared definitions, although, the tradeoff was the loss of information about supplements not listed on the card. Second, some respondents viewed themselves as having or not having a “complementary medicine identity” and this perception affected the way they interpreted and answered questions, such that response error occurred even when the term “natural herbs” was understood as intended. To mitigate this, the description of “a typical herb user’s” lifestyle was removed from the lead-in statement [10,62].

#### Perceptions of complementary therapies

A section on perceptions of complementary therapies was explored for the 2007 questionnaire. The questions were to capture respondents’ perceptions about the scientific evidence, safety, effectiveness, and holistic aspects of complementary therapies, as well as items concerning locus of control and how decisions are made to use or not use complementary therapies. The initial plan was to ask a long series of items about each individual complementary therapy, but time constraints made this unfeasible. QDRL staff then tested a version asking about complementary therapies as a whole, and determined that respondents’ views vary by specific therapies such that they could not arrive at a single answer. Ultimately the section was drastically reduced and embedded within each therapy rather than as a stand-alone section.

### 2012

#### Concepts of wellness and well-being

In fall 2010, staff from NCHS’s cognitive lab performed inductive interviews with 24 volunteers to learn more about individuals who use complementary therapies to promote their wellness, general health and well-being. The interviewer protocol contained questions to elicit reasons or motivations for using complementary therapies without presupposing it was to treat a specific condition. In particular - and in keeping with the published literature [35,36,38-40] - respondents were asked about changes noted in “mind or body” when using complementary therapies. Interviewing focused on how respondents who identified “wellness” and “well-being” as their main reason for using complementary therapies understood and conceptualized these terms. Because the intent was to learn more about a general concept or concepts rather than test draft questionnaire items, a more open-ended approach was utilized. When analyzing and interpreting the interviews, researchers aimed for “thick description,” that is, to provide a deeper, more nuanced understanding of the ways that individuals interpret events and phenomena in their social worlds. New participants were recruited and interviewed until no new conceptual insights were identified (saturation). Testing revealed that although respondents reported benefits to their physical health, they primarily associated complementary medicine use with emotional and psychological benefits. The most frequently mentioned benefits were the positive effect on one’s mood, stress level, and overall mental and emotional health. In addition to these emotional and psychological benefits, users also described many different physical benefits, most often mentioned in conjunction with their use of acupuncture, massage, or chiropractic care, and in particular, to help treat and eliminate back pain. The primary components of respondents’ conceptualizations of well-being included being centered, finding balance, and not simply being disease free, but optimally healthy [63].

Based on these qualitative research findings and related literature, a series of items for the 2012 questionnaire was drafted by NCCAM and NCHS staff to capture wellness-related reasons for using complementary therapies. Examples include “because it is natural,” and “because it focuses on the whole person, mind, body, and spirit.” Additionally, some respondents mentioned having benefited from wellness-related outcomes as a result of using complementary medicine. Hence, the questionnaire includes several items asking if using complementary medicine led to outcomes such as “improve your overall health and make you feel better,” “motivate you to eat healthier,” and “help to reduce your stress level or to relax” (Table 8).

**Table 8:** Examples of wellness-related questions deleted or modified based on cognitive review, focus groups and other input, 2012

**Question (s)**	**Outcome**	**Rationale**
*Help you relax*	**Combined** into “help to reduce your stress level or to relax”	Testing found the questions to be redundant
*Help to reduce your stress level*		
*To cleanse/detoxify your body*	**Deleted**	Respondents offered a wide variety of interpretations and meaning varied tremendously from person to person. Some considered it to be referring to “a cleanse” in the very literal sense, such as a colon cleanse. One respondent provided the example of the cayenne pepper and maple syrup diet. Others thought it referred to the period after an addict stops using drugs or alcohol and attempts to purge the body of these substances. For still others this was interpreted to be more symbolic, as in the case of a “mental or emotional cleans”’ as one respondent put it [63].
*Because the practitioner spends more time with you than medical doctors*	**Deleted**	Several respondents explained that they pay for the time with the practitioner. As one respondent put it after hearing this question, “Well, yeah, [because] I paid for 20 minutes.” Another respondent similarly explained that you “pay for the time” with the practitioner and so “of course you get more time with them” (he was thinking specifically about massage practitioners) [63].
*Because the practitioner treats the whole person and not just one part because it focuses on the whole person and not just one part*	**Combined/Modified** to “it focuses on the whole person, mind, body, and spirit”	The question did not work well when focused on “the practitioner,” especially for massage. Several noted that they actually want their masseuse to focus on only one part of, for example, their backs, and not their entire bodies. Also, the phrase “mind, body, and spirit” resonated with respondents’ experiences with complementary therapies [63].
*Because I can participate in decisions about my health with my practitioner*	**Deleted**	The question had varying interpretations and varied widely based on the type of practitioner being seen. Respondents using “hands-on” treatments such as massage said they can tell their practitioners where to focus and which parts of their bodies need more attention. Others thought it meant “sitting down with my doctor, presenting the symptoms and then working together to come up with a solution. [63]”
*To stay healthy*	**Deleted**	A number of respondents commented that this either seemed “too broad” or ‘too obvious.” As one respondent put it, “You go to a doctor to stay healthy. It’s too obvious.” Another asked, “What’s healthy? That’s too general.” [63]
Thinking about seeing a practitioner for [modality], please tell me if any of these statements are true for you. Using [modality] has…	**Modified to accommodate proxy reporting and include items salient to children**	Question stem and individual items modified to ask parent proxy whether he/she “thinks” these items applied to his/her child, and items thought to be unknown by parent were dropped. Also, the items on alcohol and smoking were not asked for children. Attendance at school was added.
Given you a sense of control over your health?		
Helped you to relax?		
Helped you to reduce your stress level?		
Motivated you to eat healthier?		
Motivated you to eat more organic foods?		
[for respondents who report drinking alcohol in core]		
Motivated you to cut back or stop drinking alcohol?		
[for respondents who report smoking in core]		
Motivated you to cut back or stop smoking cigarettes, cigars, or pipes?		
Motivated you to exercise more regularly?		
Improved your overall health and made you feel better?		
Given you more hope for the future?		
Increased your ability to focus?		
Made you feel better emotionally?		
Made it easier to cope with health problems?		
Improved your outlook on life?		
Improved your relationships with others?		
Improved your self-confidence?		
During the past 12 months, did you see a practitioner for [modality] for any of these reasons?	**Modified to accommodate proxy reporting and include items salient to children**	Question stem and individual items modified so that parent proxy could answer about his/her child, and items thought to be unknown by parents were dropped. Item on sexual performance not asked for children.
To stay healthy		
To improve your energy		
To Improve your immune function		
To improve your physical ability		
To improve your athletic or sports		
performance		
To improve your sexual performance		
To improve your concentration		
To improve your memory		
To improve your flexibility		
To improve your muscle strength		

#### Draft questionnaire

Twenty-four cognitive interviews were conducted on several different versions of the 2012 draft questionnaire in an iterative process during which revisions were made and retested. Testing focused particularly on two sections: 1) A large set of questions about insurance and payment; and 2) the expanded list of reasons for using the therapy, especially for general wellness and wellbeing. Cognitive testing and focus group feedback (see below) determined that the questions on insurance coverage of, and payment for, complementary therapies were too complex to adequately capture valid information in the limited survey time available. They were replaced with a simple question asking if health insurance covered any of the costs; if yes, did it cover all, some, or none, and how much the respondent paid in total or per visit for the particular therapy [64].

#### CAMHI conducted focus groups and quantitative analyses

To provide additional input on the 2012 questionnaire, investigators from the Child and Adolescent Health Measurement Initiative (CAMHI) based at the Oregon Health & Sciences University performed quantitative analysis of data from the 2007 child complementary medicine questionnaire including frequencies analysis of the prevalence of each therapy by age group (0–5, 6–11, 12–17), race/ethnicity (Hispanic, white non-Hispanic, black non-Hispanic, other non-Hispanic), disease status, and whether the child used any complementary medicine for reported health conditions [65]. Also explored was how these prevalence rates varied depending on which definition of complementary medicine was used (e.g., whether vitamins and minerals were included in the definition). These analyses led to the identification of sample size deficiencies, appropriateness of given therapies among different age groups, and construct validity of items related to complementary medicine use [65]. Resulting modifications to the 2012 survey included: 1) restricting the child survey to children aged 4 and older; 2) for both the child and adult questionnaires, follow-up questions were not asked for individual traditional healers, movement therapies, and special diets. Rather, individual complementary approaches within each category were grouped to increase cell sizes; and 3) follow-up questions on the reasons children or adults used Ayurveda and chelation therapy were not asked due to small cell sizes on earlier surveys.

In addition, CAHMI organized and conducted key informant interviews and focus group discussions with 21 volunteers from the Family Voices Network [66], as well as from the CAHMI network of expert advisors, pediatric provider partners and family leaders partnering on an NIH/NCCAM grant related to pediatric use of complementary therapies. Informed consent was obtained from all interview and focus group participants. Informant interview and focus group recommendations included: (1) further refinement of the cost and insurance questions, (2) inclusion of child-specific health and well-being reasons for using complementary medicine, (3) the addition of questions about complementary medicine information-seeking, (4) communication with other health care providers about complementary medicine use, and (5) the addition of an item on satisfaction of complementary medicine use for a given reason for use [65]. Although CAMHI-conducted focus groups and quantitative analyses focused on the child component, the provided input contributed to refinement of both the 2012 child and adult questionnaires.

## Discussion/conclusions

In this paper, we detail the multifaceted, largely qualitative, iterative process based on literature reviews, expert panel input, and cognitive interviewing methods used to develop and refine the NHIS complementary medicine questionnaires. For the 2012 questionnaire, we also used inductive cognitive review to conceptualize the concept of “well-being” and focus groups with family leaders and subject matter experts to further refine the child questionnaire. By soliciting feedback from subject matter experts, family leaders, and the lay public, we improved the likelihood that the questionnaire items will be understood and interpreted as intended. We describe the systematic evaluation of questionnaire items, the decision-making processes, and rationale for how items were retained, modified, or deleted. By providing this in-depth description of how these influential national surveys were developed, we hope to enable policy makers and researchers to better judge the content validity and utility of the questionnaires and the resultant findings. We also illustrate how the questionnaires have adapted over time to new scientific information and societal shifts so that modifications can be better understood by data users.

There are several limitations to the approach we used to develop the NHIS complementary medicine questionnaires. First, due to time constraints, in many cases, we were not able to continue cognitive testing until we reached saturation and may have missed some problematic issues with the questionnaires that could potentially reduce validity or generalizability. Second, although great care was taken to select individuals for cognitive testing who differed widely in demographic characteristics, the volunteers may not have been representative of the U.S. population in all important respects, which could limit generalizability of the testing results. Third, we did not include lay people on the expert panels convened to determine content areas for the questionnaires and thus could have missed salient topics of particular interest to users of complementary medicine. However, lay public were included in the CAHMI-organized focus groups that identified additional survey topics not prioritized by the think tank experts. Fourth, there is no universally accepted definition of what constitutes a complementary medicine approach. As a result, there is no definitive list of which therapies should be included in the questionnaire. For instance, whether prayer for health reasons should be considered within the realm of complementary therapies has received considerable discussion [67,68]. For these surveys, we have been as inclusive as possible given time constraints, thus allowing greatest flexibility to data users. Fifth, the NHIS has limitations common to all cross-sectional studies collecting participant-reported data: it is not possible to make inferences about cause and effect, and data are not independently verified. This latter limitation may be particularly important for the section on out-of-pocket expenditures in which respondents likely provide estimates versus exact amounts. Finally, in the 2007 survey and for some respondents in the 2012 survey, although the cost per visit or purchase was collected as continuous data within the range of $0-$500 per expenditure, the number of visits or purchases per person was collected as categorical data (based on recommendations from cognitive testing). This mixing of continuous and categorical data makes the computation of actual expenditures on complementary therapies very complex [69]. For the 2012 survey, we hope to provide better guidance and technical documents explaining how to analyze the expenditure data.

The three NHIS complementary medicine questionnaires were developed to assess the U.S. public’s use of complementary health approaches and factors thought to be associated with this use. It is our expectation that quantitative analyses on the validity and reliability of the questionnaires will supplement the qualitative development approaches describe here, and will inform future survey design and provide guidance to users of existing survey data. Through changes over time, the NHIS complementary medicine surveys have attempted to meet the challenge of providing information to the NCCAM/NIH, national academies, researchers, clinicians, and policy makers. The NHIS complementary medicine datasets remain the primary national source of data on complementary medicine use in the United States.

## Abbreviations

NHIS: National Health Interview Survey; CDC: Centers for disease control and prevention; NIH: National institutes of health; NCCAM: National center for complementary and alternative medicine; NVNMDS: Nonvitamin, nonmineral dietary supplements; NCHS: National center for health statistics; QDRL: Questionnaire design research laboratory; CAMHI: Oregon Health & Sciences University’s Child and Adolescent Health Measurement Initiative (CAMHI).

## Competing interests

The authors declare that they have no competing interests.

## Authors’ contributions

BJS participated in survey design, data analysis, and drafted substantial portions of the manuscript. CDB participated in survey design, data analysis, and drafted substantial portions of the manuscript. CG participated in data analysis and revised substantial portions of the manuscript. RLN participated in survey design, and drafted substantial portions of the manuscript. All authors read and approved the final manuscript.

## Pre-publication history

The pre-publication history for this paper can be accessed here:

http://www.biomedcentral.com/1472-6882/13/328/prepub

## References

[B1] National Center for Complementary and Alternative Medicine Expanding Horizons of Healthcare Five-Year Strategic Plan 2001–2005 2000 Bethesda, Maryland, USA: US Department of Health and Human Services, National Institutes of Health, National Center for Complementary and Alternative Medicine NIH Publication number 01–5001

[B2] Publications Using NHIS CAM Datahttp://nccam.nih.gov/news/camstats/2007/bibliography.htm

[B3] National Center for Complementary and Alternative Medicine Exploring the Science of Complementary and Alternative Medicine Third Strategic Plan: 2011–2015 2011 Bethesda, Maryland, USA: US Department of Health and Human Services, National Institutes of Health, National Center for Complementary and Alternative Medicine NIH Publication number 11–7643, D458

[B4] Veale B Questionnaire design and surveys Aust Fam Physician 1998 13 499 502 9648317

[B5] Passmore C Dobbie AE Parchman M Tysinger J Guidelines for constructing a survey Fam Med 2002 13 281 286 12017142

[B6] Rattray J Jones MC Essential elements of questionnaire design and development J Clin Nurs 2007 13 234 243 Review 17239058 10.1111/j.1365-2702.2006.01573.x

[B7] Cella D Riley W Stone A Rothrock N Reeve B Yount S Amtmann D Bode R Buysse D Choi S Cook K Devellis R DeWalt D Fries JF Gershon R Hahn EA Lai JS Pilkonis P Revicki D Rose M Weinfurt K Hays R PROMIS Cooperative Group The Patient-Reported Outcomes Measurement Information System (PROMIS) developed and tested its first wave of adult self-reported health outcome item banks:2005–2008 J Clin Epidemiol 2010 13 1179 1194 10.1016/j.jclinepi.2010.04.011 20685078 PMC2965562

[B8] DeWalt DA Rothrock N Yount S Stone AA PROMIS Cooperative Group Evaluation of item candidates: the PROMIS qualitative item review Med Care 2007 13 Suppl 5 12 21 10.1097/01.mlr.0000254567.79743.e2 PMC2810630 17443114

[B9] Vital and Health Statistics Questionnaire Design in the Cognitive Laboratory 1989 13 Hyattsville, Maryland, USA: NCHS DHHS Publication No. (PHS) 89–1076

[B10] Willson S Stussman B Maitland A Nahin RL Role of self-concept in answering survey questions on complementary and alternative medicine: challenges to and strategies for improving data quality J Altern Complement Med 2009 13 1319 1325 10.1089/acm.2008.0580 19954343

[B11] Wootton JC Sparber A Surveys of complementary and alternative medicine: part I. General trends and demographic groups J Altern Complement Med 2001 13 195 208 10.1089/107555301750164307 11327525

[B12] The Dietary Supplement Health and Education Act of 1994 (Public Law 103–417, DSHEA) http://ods.od.nih.gov/About/MissionOriginMandate.aspx

[B13] Ajazuddin Saraf S Legal regulations of complementary and alternative medicines in different countries Pharmacogn Rev 2012 13 154 160 23055642 10.4103/0973-7847.99950 PMC3459458

[B14] Watanabe S Imanishi J Satoh M Ozasa K Unique place of Kampo (Japanese traditional medicine) in complementary and alternative medicine: a survey of doctors belonging to the regional medical association in Japan Tohoku J Exp Med 2001 13 55 63 10.1620/tjem.194.55 11556734

[B15] Eisenberg DM Kessler RC Foster C Norlock FE Calkins DR Delbanco TL Unconventional medicine in the United States. Prevalence, costs, and patterns of use N Engl J Med 1993 13 246 252 10.1056/NEJM199301283280406 8418405

[B16] Paramore LC Use of alternative therapies: estimates from the 1994 Robert Wood Johnson Foundation National Access to Care Survey J Pain Symptom Manage 1997 13 83 89 10.1016/S0885-3924(96)00299-0 9095565

[B17] Astin JA Why patients use alternative medicine: results of a national study JAMA 1998 13 1548 1553 10.1001/jama.279.19.1548 9605899

[B18] Eisenberg DM Davis RB Ettner SL Appel S Wilkey S Van Rompay M Kessler RC Trends in alternative medicine use in the United States, 1990–1997: results of a follow-up national survey JAMA 1998 13 1569 1575 10.1001/jama.280.18.1569 9820257

[B19] Druss BG Rosenheck RA Association between use of unconventional therapies and conventional medical services JAMA 1999 13 651 656 10.1001/jama.282.7.651 10517718

[B20] Burg MA Hatch RL Neims AH Lifetime use of alternative therapy: a study of Florida residents South Med J 1998 13 1126 1131 10.1097/00007611-199812000-00006 9853724

[B21] Oldendick R Coker AL Wieland D Raymond JI Probst JC Schell BJ Stoskopf CH Population-based survey of complementary and alternative medicine usage, patient satisfaction, and physician involvement. South Carolina Complementary Medicine Program Baseline Research Team South Med J 2000 13 375 381 10798505

[B22] Ni H Simile C Hardy AM Utilization of complementary and alternative medicine by United States adults: results from the 1999 National Health Interview Survey Med Care 2002 13 353 358 10.1097/00005650-200204000-00011 12021691

[B23] Mackenzie ER Taylor L Bloom BS Hufford DJ Johnson JC Ethnic minority use of complementary and alternative medicine (CAM): a national probability survey of CAM utilizers Altern Ther Health Med 2003 13 50 56 12868252

[B24] Barnes PM Powell-Griner E McFann K Nahin RL Complementary and alternative medicine use among adults: United States, 2002 Adv Data 2004 13 1 19 15188733

[B25] Upchurch DM Chyu L Use of complementary and alternative medicine among American women Womens Health Issues 2005 13 5 13 10.1016/j.whi.2004.08.010 15661582

[B26] Honda K Jacobson JS Use of complementary and alternative medicine among United States adults: the influences of personality, coping strategies, and social support Prev Med 2005 13 46 53 10.1016/j.ypmed.2004.05.001 15530580

[B27] Rafferty AP McGee HB Miller CE Reyes M Prevalence of complementary and alternative medicine use: state-specific estimates from the 2001 Behavioral Risk Factor Surveillance System Am J Public Health 2002 13 1598 1600 10.2105/AJPH.92.10.1598 12356602 PMC1447288

[B28] Carlson MJ Krahn G Use of complementary and alternative medicine practitioners by people with physical disabilities: estimates from a National US Survey Disabil Rehabil 2006 13 505 513 10.1080/09638280500212062 16513583

[B29] Wilson KM Klein JD Sesselberg TS Yussman SM Markow DB Green AE West JC Gray NJ Use of complementary medicine and dietary supplements among U.S. adolescents J Adolesc Health 2006 13 385 394 10.1016/j.jadohealth.2005.01.010 16549299

[B30] Bair YA Gold EB Zhang G Rasor N Utts J Upchurch DM Chyu L Greendale GA Sternfeld B Adler SR Use of complementary and alternative medicine during the menopause transition: longitudinal results from the Study of Women’s Health Across the Nation Menopause 2008 13 32 43 10.1097/gme.0b013e31813429d6 18090037

[B31] Hsiao AF Wong MD Goldstein MS Yu HJ Andersen RM Brown ER Becerra LM Wenger NS Variation in complementary and alternative medicine (CAM) use across racial/ethnic groups and the development of ethnic-specific measures of CAM use J Altern Complement Med 2006 13 281 290 10.1089/acm.2006.12.281 16646727

[B32] Harrigan R Mbabuike N Efird JT Easa D Shintani T Hammatt Z Perez J Shomaker TS Use of provider delivered complementary and alternative therapies in Hawai’i: results of the Hawai’i Health Survey Hawaii Med J 2006 13 130 132 134–139, 151 16774141 PMC1934565

[B33] Cheung CK Wyman JF Halcon LL Use of complementary and alternative therapies in community-dwelling older adults J Altern Complement Med 2007 13 997 1006 10.1089/acm.2007.0527 18047447

[B34] Smith TC Ryan MA Smith B Reed RJ Riddle JR Gumbs GR Gray GC Complementary and alternative medicine use among US Navy and Marine Corps personnel BMC Complement Altern Med 2007 13 7 16 10.1186/1472-6882-7-16 PMC1884175 17506899

[B35] McCaffrey AM Pugh GF O’Connor BB Understanding patient preference for integrative medical care: results from patient focus groups J Gen Intern Med 2007 13 1500 1505 10.1007/s11606-007-0302-5 17846846 PMC2219802

[B36] Green AM Walsh EG Sirois FM McCaffrey A Perceived benefits of complementary and alternative medicine: a whole systems research perspective Open Comp Med J 2009 13 35 45

[B37] Wolsko PM Eisenberg DM Davis RB Ettner SL Phillips RS Insurance coverage, medical conditions, and visits to alternative medicine providers: results of a national survey Arch Intern Med 2002 13 281 287 10.1001/archinte.162.3.281 11822920

[B38] Vincent C Furnham A Willsmore M The perceived efficacy of complementary and orthodox medicine in complementary and general practice patients Health Educ Res 1995 13 395 405 10159673 10.1093/her/10.4.395-a

[B39] McGregor KJ Peay ER The choice of alternative therapy for health care: testing some propositions Soc Sci Med 1996 13 1317 1327 10.1016/0277-9536(95)00405-X 8913002

[B40] Swartzman LC Harshman RA Burkell J Lundy ME What accounts for the appeal of complementary/alternative medicine, and what makes complementary/ alternative medicine “alternative”? Med Decis Making 2002 13 431 450 12365485 10.1177/027298902236943

[B41] Nutrition Business Journal Supplement Business Report 2010 2010 Boulder, CO: New Hope Natural Media, Penton Media, Inc

[B42] Fowler F Improving Survey Questions 1995 Thousand Oaks, California: Sage Publications

[B43] Tourangeau R Rips L Rasinski K The Psychology of Survey Response 2000 Cambridge, New York: Cambridge University Press

[B44] Meadows KA So you want to do research? 5: questionnaire design Br J Community Nurs 2003 13 562 570 14688664 10.12968/bjcn.2003.8.12.11854

[B45] Smith BJ Tang KC Nutbeam D WHO Health Promotion Glossary: new terms Health Promot Int 2006 13 340 345 10.1093/heapro/dal033 16963461

[B46] Diener E Subjective well-being Psychol Bull 1984 13 542 572 6399758

[B47] Ryff CD Happiness is everything, or is it? Explorations on the meaning of psychological well-being J Pers Soc Psychol 1989 13 1069 1081

[B48] Bufford RK Paloutzian RF Ellison CW Norms for the spiritual well-being scale J Psychol Theology 1991 13 56 70

[B49] Ware JE Jr Sherbourne CD The MOS 36-item short-form health survey (SF-36). I. Conceptual framework and item selection Med Care 1992 13 473 483 10.1097/00005650-199206000-00002 1593914

[B50] McHorney CA Ware JE Jr Raczek AE The MOS 36-item short-form health survey (SF-36). II. Psychometric and clinical tests of validity in measuring physical and mental health constructs Med Care 1993 13 247 263 8450681 10.1097/00005650-199303000-00006

[B51] McHorney CA Ware JE Jr Lu JF Sherbourne CD The MOS 36-item short-form health survey (SF-36). III. Tests of data quality, scaling assumptions, and reliability across diverse patient groups Med Care 1994 13 40 66 10.1097/00005650-199401000-00004 8277801

[B52] Adams T Bezner J Steinhardt M The conceptualization and measurement of perceived wellness: integrating balance across and within dimensions Am J Health Promot 1997 13 208 218 10.4278/0890-1171-11.3.208 10165100

[B53] Hatch RL Burg MA Naberhaus DS Hellmich LK The spiritual involvement and beliefs scale. Development and testing of a new instrument J Fam Pract 1998 13 476 486 9638112

[B54] Ryan RM Deci E On happiness and human potentials: a review of research on hedonic and eudaimonic well-being Annu Rev Psychol 2001 13 141 166 10.1146/annurev.psych.52.1.141 11148302

[B55] Gomez R Fisher JW Domains of spiritual well-being and development and validation of the spiritual well-being questionnaire Personal Individ Differ 2003 13 1975 1991 10.1016/S0191-8869(03)00045-X

[B56] Gallager MW The hierarchical structure of well-being J Pers 2009 13 1025 1049 10.1111/j.1467-6494.2009.00573.x 19558444 PMC3865980

[B57] Harris-Kojetin LD Fowler FJ Jr Brown JA Schnaier JA Sweeny SF The use of cognitive testing to develop and evaluate CAHPS 1.0 core survey items. Consumer Assessment of Health Plans Study Med Care 1999 13 Suppl 3 10 21 10.1097/00005650-199903001-00002 10098555

[B58] Ospina MB Bond TK Karkhaneh M Meditation Practices for Health: State of the Research. Evidence Report/ Technology Assessment no. 155 2007 Rockville, MD: Agency for Healthcare Research and Quality AHRQ publication no. 07-E010 PMC4780968 17764203

[B59] Beatty P Willis G Research synthesis: the practice of cognitive interviewing Public Opin Q 2007 13 287 311 10.1093/poq/nfm006

[B60] Corbin J Strauss A Basics of Qualitative Research 3e 2008 Thousand Oaks, CA: Sage Publications, Inc

[B61] Miller K Cognitive Interview Evaluation of the Complementary and Alternative Medicine Supplement for the National Health Interview Survey: Results of Interviews conducted June-July, 2001 2001 Hyattsville, MD: National Center for Health Statistics

[B62] Willson S Cognitive Interviewing Evaluation of the 2007 Complementary and Alternative Medicine Module for the National Health Interview Survey. Interviews conducted February 13-March 1 and May 1-May 12 2006 2006 Hyattsville, MD: National Center for Health Statistics

[B63] Gray C Chepp V Irby A Report on Complementary and Alternative Medicine and Well-Being Study conducted at the National Center for Health Statistics, QDRL. Interviews conducted September 1-October 6 2010 2010 Hyattsville, MD: National Center for Health Statistics

[B64] Gray C Chepp V Cognitive Interview Evaluation of the Complementary and Alternative Medicine Supplement for the National Health Interview Survey: Results of Interviews conducted February-April, 2011 2011 National Center for Health Statistics: Hyattsville, MD

[B65] Bethell C Gombojav N Stumbo S Brown C Blumberg S Carle A Newacheck PW Using the National Health Interview Survey and Medical Expenditures Panel Survey to Assess the Use and impact of Complementary and Alternative Medicine for Children in the US: A Meta ‒Data Methods Key Issues Report 2013 Portland, Oregon, USA: The Child and Adolescent Health Measurement Initiative

[B66] Family Voices Networkhttp://www.familyvoices.org/projects?id=0003. Last accessed October 25, 2013

[B67] Tippens K Marsman K Zwickey H Is prayer CAM? J Alt Comp Med 2009 13 435 438 10.1089/acm.2008.0480 PMC3004781 19388867

[B68] Kaptchuk TJ Eisenberg DM Varieties of healing. 2: a taxonomy of unconventional healing practices Annals Int Med 2001 13 196 204 10.7326/0003-4819-135-3-200108070-00012 11487487

[B69] Nahin RL Barnes PM Stussman BJ Bloom B Costs of complementary and alternative medicine (CAM) and frequency of visits to CAM practitioners: United States, 2007 Natl Health Stat Report 2009 13 1 14 19771719

